# Bioflavonoid Robinin from *Astragalus falcatus* Lam. Mildly Improves the Effect of Metothrexate in Rats with Adjuvant Arthritis

**DOI:** 10.3390/nu13041268

**Published:** 2021-04-13

**Authors:** Lia Tsiklauri, Karol Švík, Martin Chrastina, Silvester Poništ, František Dráfi, Lukáš Slovák, Mery Alania, Ether Kemertelidze, Katarina Bauerova

**Affiliations:** 1 Iovel Kutateladze Pharmacochemistry Institute, Tbilisi State Medical University, P. Sarajishvilist 36, Tbilisi 0159, Georgia; l.tsiklauri@tsmu.edu (L.T.); m.alania@tsmu.edu (M.A.); e.kemertelidze@tsmu.edu (E.K.); 2 Centre of Experimental Medicine, Institute of Experimental Pharmacology and Toxicology, Slovak Academy of Sciences, Dúbravská cesta 9, 84104 Bratislava, Slovakia; karol.svik@savba.sk (K.Š.); martin.chrastina@savba.sk (M.C.); silvester.ponist@savba.sk (S.P.); frantisek.drafi@savba.sk (F.D.); lukas.slovak@savba.sk (L.S.); 3 Jessenius Faculty of Medicine in Martin, Malá Hora 4A, 03601 Martin, Slovakia

**Keywords:** arthritis, flavonoids, robinin, methotrexate, *astragalus*

## Abstract

Anti-inflammatory potential of orally administrated bioflavonoid-robinin, active sub-stance of original drug Flaroninum™ (FL), was investigated in the combination with methotrexate (MTX) and in monotherapy in rats suffering from adjuvant-induced arthritis (AA). Robinin (kaempferol-3-*O*-robinoside-7-*O*-rhamnoside) was isolated from the aerial parts of *Astragalus falcatus* Lam. The monotherapy with robinin was not efficient in alleviating symptoms of AA. The combination of MTX with robinin was similarly active as MTX alone in reducing the hind paw volume and change of body weight during the whole experiment. The combination, however, reduced plasma levels of Interleukin-17Aand activity of gamma-glutamyl transferase in joint more efficiently then MTX alone. Our results demonstrate that the novel combination of robinin and MTX mildly improved the reduction of inflammation in experimental arthritis.

## 1. Introduction

Rheumatoid arthritis (RA) is an autoimmune inflammatory disease, which preferably targets the synovial joints and is associated with premature mortality and socioeconomic burdens [1]. The pathogenesis of RA is not fully understood yet, it is suggested that the abnormal activation of inflammatory signaling pathways, hormonal imbalance, smoking, and oxidative stress play significant roles in the development of RA [2]. Current treatment strategies for RA are based on the aim to control disease activity, lower structural damages, and enhance quality of life. In addition to conventional disease-modifying antirheumatic drugs (DMARDs), that are particularly associated with different side effects, targeted DMARDs are more suitable for the treatment of RA [3,4]. Clinical studies showed that combinations of drugs are more effective than a single medicine alone for the management of inflammatory arthritis. Methotrexate (MTX) is the first line drug in the treatment of rheumatoid arthritis [5]. Natural remedies became a significant field of interest for the development of newer medicines, because they are a rich source of different compounds with potentially novel mechanisms of action. Indeed, anti-inflammatory phytomolecules were shown to possess a wide scale mechanism of action including interactions with the inflammatory pathways [6,7]. Robinin belongs to flavonoids, which are an essential group of naturally occurring polyphenolic compounds, widely distributed in the plant kingdom and exhibiting vast range of beneficial biological activities, including antiphlogistic (anti-inflammatory) action. These effects may interact directly with pro-inflammatory signal proteins. Flavonoids may affect specifically the function of enzyme systems critically involved in the generation of inflammatory processes, especially tyrosine and serine-threonine protein kinases [8,9]. However, the use of these compounds in clinical praxis is modest due to their low bioavailability [10]. In our experiment, we selected the higher dose of robinin from our pilot study with robinin applied in two doses (unpublished results) to partially compensate it’s low bioavailability.

**Figure 1:**
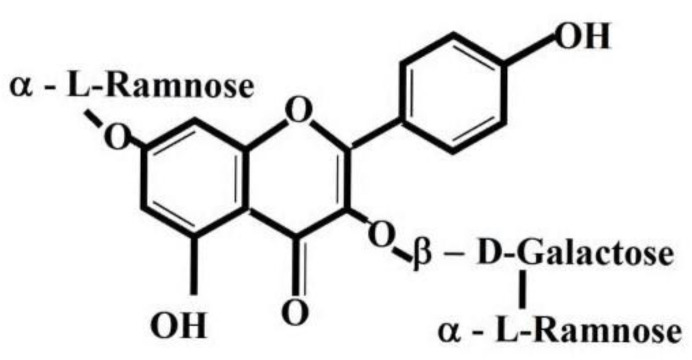
Chemical structure of kaempferol 3-*O*-robinoside-7-*O*-rhamnoside (robinin (RB)).

Robinin is found in plants of the genus *Astragalus* which is broadly distributed throughout the temperate and arid regions and known to contain phytochemicals, which are found in most parts of the plant and have been associated with multiple actions i.e., anti-inflammatory, pain relieving and protective effects [11,12]. *Astragalus falcatus* Lam. is widely distributed in Georgia. This plant has been intensively investigated at the I. Kutateladze Pharmacochemistry Institute (IKPI) of the Tbilisi State Medical University (TSMU) and hypoazotemic flavonoid glycoside robinin was isolated from its aerial parts [13,14]. Based on this compound, the original preparation Flaroninum™ (FL) in the form of tablets has been proposed [14]. FL has been approved as a drug by the Georgian and Russian healthcare authorities. This medicine possess diuretic and hypotensive effects. The active substance of this product is robinin, which is one of the derivatives of kaempferol (Figure 1). This substance has been isolated also from plants *Pueraria hirsuta* (Thunb.) Matsun. and *Astragalus* sp. [15,16]. The bioflavonoid robinin (RB) exerts anti-inflammatory and strong antioxidant activities. Eom et al. [17] have reported Kudzu leaf extract, with main constituent robinin, reduced the expression of inducible nitric oxide synthase, cyclooxygenase-2, interleukin-6, and tumor necrosis factor-α. Robinin therapeutically improved lipid peroxidation in heart tissue and inflammatory markers, cyclooxygenase-2 and lipoxygenase-15 and reduced cardiotoxicity by modulating transforming growth factor beta 1signaling pathway in Sprague Dawley rats [18]. Robinin inhibited the oxidized low-density lipoprotein (LDL) lipoprotein (ox-LDL) induced toll-like receptors 2 (TLR2) and toll-like receptors 4 (TLR4) expression and inhibited the translocation of nuclear factor kappa B (NF-κB) p65 by modulating the TLR-NF-κB signaling pathway [19]. In this study, we examined the potential anti-inflammatory properties of orally administrated robinin in the experimentally-induced arthritis in monotherapy and in combination therapy with MTX. In this context, we used rat adjuvant induced arthritis (AA) model: This well-known model is T-lymphocyte dependent and is associated with hyperplasia of the synovium and inflammation in joints [20,21].

## 2. Materials and Methods

Flavonoid glycoside robinin (≥95%) was isolated at IKPI. Flowers and leaves of *Astragalus falcatus* Lam. were collected from Didi Toneti, Kartli floristic region, Georgia, and identified by botanists of Pharmacobotany, Department of Phytochemistry of IKPI. Voucher specimens (N 16354) were deposited at the herbarium collection of IKPI (Herbarium Code-TBPH). Air dried and crushed aerial parts (leaves and flowers) (10 kg) of *Astragalus falcatus* Lam. were extracted with 70% ethanol three times (raw material:solvent, 1:10, *w*/*v*; at room temperature) by maceration for 3 days. The combined extracts were dried under low pressure (100–140 mm Hg,) at 70–80 °C to obtain 15 L of aqueous residue. The remaining aqueous phase was filtered and treated with chloroform (1 L) 3 times to remove lipophilic compounds; then, a small volume of organic solvent (chloroform) was added and kept for 24 h. Formed crystals were filtered, washed with ethanol (45%), and air dried. This technical product was recrystallized from diluted ethanol (45%) and dried under vacuum at 70–80 °C; the yield of obtained robinin was 1.72%. The flavonoid content was analyzed by spectrophotometric method using aluminum chloride. In brief, the stock solution of robinin (0.2%) was prepared in ethanol (70%) by gentle heating. The work solution (0.004%) was obtained by appropriate dilution of stock solution with 70% ethanol. AlCl_3_ solution (5 mL, 0.5%, *w*/*v*) was added to 5 mL of this (or standard 0.004%) solution and mixed. Then, after 10 min of incubation at room temperature, samples were subjected to spectral analysis. Absorbance measurements were performed on the spectrophotometer using 10 mm width cuvette against a blank solution (which contained equal volumes (5 mL) of ethanol (70%) and aluminium chloride (0.5%) solutions) at 390 nm. The concentration of standard solution of robinin was 0.004%. Robinin standard (99% purity) was obtained in Phenolic Compounds, Department of Phytochemistry of IKPI. For this experiment, the stock suspension of tested compound was prepared on daily basis, as vehiculum redistilled water was used. The incomplete Freund’s adjuvant (IFA) was obtained from MERCK (Sigma Aldrich, St. Louis, MO, USA) and heat-inactivated *Mycobacterium butyricum* (MB) was obtained from BD Difco™ (Sparks, MD, USA). Methotrexate (Ebewesol^®^ inj. 20 mg/mL), heparin (HEPARIN LÉČIVA^®^ Praha, Czech Republic), tiletamine with zolazepam (Zoletil^®^ 50, Virbac, Carros, France), and xylazine (Xylariem^®^ 20 mg/mL, ECUPHAR N.V., Oostkamp, Belgium) were used for this experiment. Chemicals for γ-glutamyltransferase (GGT): buffer (2.6 mM NaH_2_PO_4_, 50 mM of Na_2_HPO_4_, 15 mM, ethylenediaminetetraacetic acid (EDTA), 68 mM NaCl; pH 8.1), 8.7 mM L-γ-glutamyl-*p*-nitroanilide, 44 mM methionine, 65% isopropyl alcohol, methanol were obtained from MERCK (Sigma Aldrich, St. Louis, MO, USA).

### 2.1. Laboratory Animals

In this experiment Lewis male rats were purchased from Department of Toxicology and Laboratory Animal Breeding, Centre of Experimental Medicine, SAS, Dobrá Voda, Slovak Republic (SK CH 24016). Immediately after housing of animals, rat were submitted to a seven-day quarantine. Animals had unlimited access to standard diet and tap water ad libitum, as well as dark/light regime 12 h/12 h. The animal housing agrees with EU Convention for the Protection of Vertebrate Animals Used for Experimental and Other Purposes. The authorization of the protocol for this experiment was done by the Ethics Committee of the Institute of Experimental Pharmacology and Toxicology, Center of Experimental Medicine SAS in Bratislava, Slovakia (SK UCH 04018) and State Veterinary and Food Administration of the Slovak Republic, Bratislava (3144/16-221/3).

### 2.2. Adjuvant Induced Arthritis in LEWIS Rats

The AA is a well-established model of inflammation [20,21]. The adjuvant arthritis was induced in rats with weight of 160–180 g (6 weeks) by an individual intradermal immunization at the base of the tail with suspension of 0.1 mL of 12 mg/mL heat-inactivated MB powder suspended in incomplete IFA according to our previous protocol [22,23].

### 2.3. Treatments and Design of Experiment

The animals were assigned to 5 groups of eight rats in each. Group one was used as healthy controls. The second was untreated AA group. The remaining three groups were AA rats treated as given in the study design below (Table 1).

The tested substance and MTX were administered orally (via gastric tube) during the whole experiment (28 days); RB was partially dissolved in tap water and administered daily; anti-rheumatic drug-MTX was diluted with tap water and applied two times a week in dosage of 0.3 mg/kg of b.w. The b.w. of animals was measured before administration of tested substances. On days 14 and 21, the blood was withdrawn into heparanized tubes from the rat’s retro-orbital plexus using tiletaminum, zolazepamum plus xylazine anesthesia. On the last experimental day, rats were sacrificed in anesthesia, the blood was obtained, and tissues (spleen and hind paw joint) were collected from all animals. Blood samples were centrifugated at 2000× *g* for 15 min. This procedure depletes the platelets from the samples of plasma. Samples were saved under −70 °C.

### 2.4. Evaluation of Experimental Arthritis

On the day 14th, 21st, and 28th after immunization of the animals, the hind paw joints were assessed. Hind paw volume (HPV) was expressed as the average of the elevation of percentage (%) of the hind paw volume of every rat, compared with HPV measured at day 1 using a water plethysmometer (UGO BASILE, Comerio-Varese, Italy). The HPV on the selected day was divided by the HPV on day 1 and expressed in per cent according to the following formula w: ([n Day]/[Day 1]) × 100 − 100 = value [%]. The body weight of the animals was measured daily. The changes in body weight are shown as average of the weight gain [g]. Weight measured on the day (n—day 14, 21 and 28) minus weight measured on day 1. The mathematical formula we used is as follows: [n Day] − [Day 1] = value [g].

### 2.5. The Activity of γ-Glutamyltransferase in the Hind Paw Joint and Spleen Tissue

The cellular activity of γ-glutamyltransferase (GGT) was measured on day 28 in the spleen and hind paw joint tissue homogenates using the method of Orlowski and Meister [24] and modified by Ondrejickova et al. [25]. The tissues were homogenized in a buffer (2.6 mM of NaH_2_PO_4_, 50 mM of Na_2_HPO_4_, 68 mM of NaCl,15 mM of EDTA; pH 8.1) at 1:9 (*w*/*v*) by Ultra Turax TP 18/10 (Janke and Kunkel) for one min at 0 °C. The biochemical substrates (44 mM of methionine and 8.7 mM of L-γ-glutamyl-*p*-nitroanilide) were dissolved in isopropyl alcohol (65%) to final concentrations of 2.5 mM and 12.6 mM, respectively. After one hour incubation at 37 °C, the reaction was stopped by adding 2.3 mL of cold methanol. Tubes were centrifuged at 5000 rpm for 20 min (Centrifuge Eppendorf). Supernatant’s absorbance (product *p*-nitroaniline) was determined on spectrophotometer Specord 40 at 406 nm. Solution mix without or without substrate or acceptor were used as blanks.

(1) $$a\;=\;\frac{A}{k}\;\times \;\frac{3.2}{0.5}\;\times \;\frac{1}{13,813}\;\times \;\frac{1000}{\mathrm{tissue}\;\left[\mathrm{mg}\right]}\;\times \;\frac{1}{60}\;\times \;1000\;\left[\;\mathrm{nmol}\;p-\mathrm{nitroaniline}/\min/\mathrm{gtissue}\right].$$

Mathematical formula explanatory note:

A—absorbance;
c—concentration of p-nitroaniline [µg/mL];
k—coefficient from calibration curve A = k. c.

### 2.6. Levels of Interleukin 17A in Plasma Samples

The level of plasmatic Interleukin-17A (IL-17A) was determined using an enzyme-linked immunosorbent assay type kit (eBioscience^®^; Waltham, MA, USA) according to the instruction of the manufacturer. *Statistical analyses.* The average values ± SEM were calculated. Significant differences between control animals, untreated animals, and treated groups of animals were determined by ANOVA. The post hoc test (Tukey-Kramer) was applied in cases where differences between groups was significant. The levels of significance after the post hoc screening were specified as followed: not significant (*p* > 0.05), significant (*p* ≤ 0.05), very significant (*p* ≤ 0.01), and highly significant (*p* ≤ 0.001).

## 3. Results

### 3.1. Change of the Body Weight (CBW)

**Figure 2:**
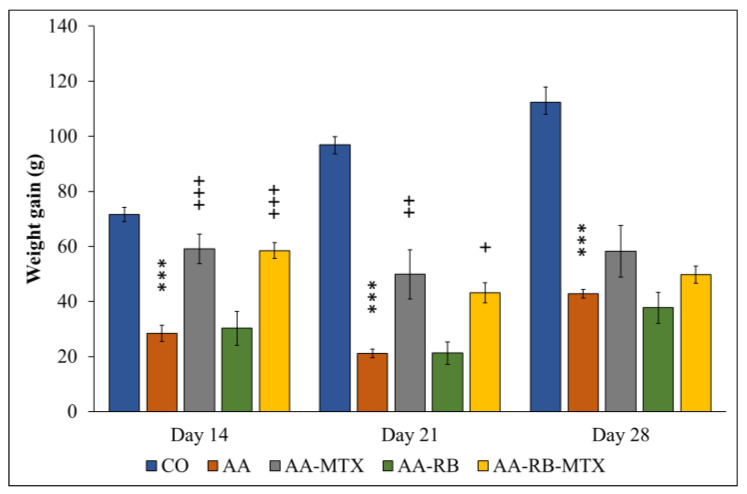
The effect of RB administered alone or in combination with MTX on the change of the body weight on days 14, 21 and 28. CO—control animals, AA—adjuvant arthritis controls, AA-MTX—adjuvant arthritis group treated with methotrexate 0.3 mg/kg of b.w. twice a week, AA-RB—adjuvant arthritic animals treated by RB in dosage of 50 mg/kg, AA-RB-MTX—adjuvant arthritic animals treated with RB (50 mg/kg) and 0.3 mg/kg of b.w. twice a week. Results were expressed as mean ± SEM, *n* = 8. Significant difference: *** *p* < 0.001 AA vs. CO, +++ *p* < 0.001 treated groups vs. AA, ++ *p* < 0.01 treated groups vs. AA, + *p* < 0.05 treated groups vs. AA. Weight measured on the day (n–day 14, 21, and 28) minus weight measured on day 1. The mathematical formula we used is as follows: [n Day] − [Day 1] = value [g].

AA animals have significantly lower weight gains in comparison to the controls. This effect was observed within the duration of the whole experiment. RB had no effect on this parameter at any analyzed day. MTX alone was partly active at day 14 and 21. Similarly, the combination decreased the loss of weight versus the control group at the same days. At the end of the experiment, however, none of the treatment significantly modified this parameter (Figure 2).

#### Hind Paw Volume (HPV)

**Figure 3:**
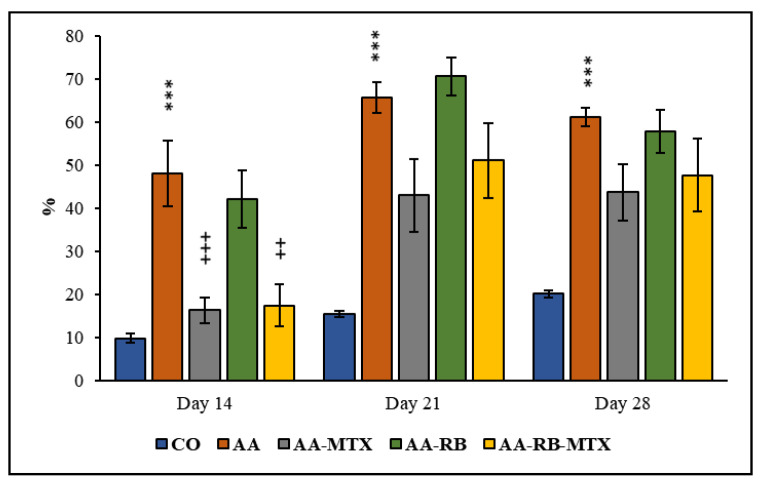
The effect of RB administered alone or in combination with MTX on hind paw volume (HPV) on days 14, 21, and 28. CO—control animals, AA—adjuvant arthritis controls, AA-MTX—adjuvant arthritis group treated with methotrexate 0.3 mg/kg of b.w. twice a week, AA-RB—adjuvant arthritic animals treated by RB in dosage of 50 mg/kg, AA-RB-MTX—adjuvant arthritic animals treated with RB (50 mg/kg) and 0.3 mg/kg of b.w. twice a week. Results were expressed as mean ± SEM, *n* = 8. Significant difference: *** *p* < 0.001 AA vs. CO, +++ *p* < 0.001 treated group vs. AA, ++ *p* < 0.01 treated group vs. AA. The HPV volume on the selected day (n—day 14, 21, and 28) is divided by the HPV volume on day 1. All this is multiplied by 100 and, finally, minus 100, we get the percentage. The mathematical formula we used is as follows: ([n Day]/[Day 1]) × 100 − 100 = value [%].

Hind paw swelling is a consequence of inflammatory, as well as arthritic, changes occurring in AA rats. AA animals have significantly higher hind paw volumes in comparison to the controls (Figure 3). This effect was observed within the duration of the whole experiment. RB had no effect on HPV at any day measured. MTX alone was significantly active in reduction of HPV at day 14. However, on days 21 and 28, the effect of MTX was only partial. Similar trend was observed with the combination being significantly active in reduction of HPV at day 14, but, on days 21 and 28, only moderate effect was measured (Figure 3).

### 3.2. Activity of Cellular γ-Glutamyl-Transferase in the Hind Paw Joint and Spleen Tissue

**Figure 4:**
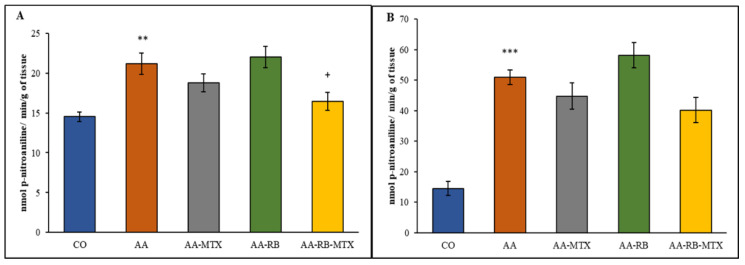
The effect of RB administered alone or in combination with MTX on the activity of GGT in joint (**A**), spleen (**B**) on day 28. CO—control animals, AA—adjuvant arthritis controls, AA-MTX—adjuvant arthritis group treated with methotrexate 0.3 mg/kg of b.w. twice a week, AA-RB—adjuvant arthritic animals treated by RB in dosage of 50 mg/kg, AA-RB-MTX—adjuvant arthritic animals treated with RB (50 mg/kg) and 0.3 mg/kg of b.w. twice a week. Results were expressed as mean ± SEM, *n* = 8. Significant difference: *** *p* < 0.001 AA vs. CO; ** *p* < 0.01 AA vs. CO, + *p* < 0.05 treated group vs. AA. The activity of cellular γ-glutamyltransferase was calculated according to the formula already mentioned in Section 2.5.

Differences in enzymatic activity of GGT in spleen and joint are shown on Figure 4 on day 28. GGT increased by 46% in joint and 250% in spleen compared with the healthy control group (Figure 4A). The MTX administration caused an insignificant reduction of GGT activity in both tissues. Application of RB did not influence, as well, the activity of GGT in both tissues (Figure 4A), but the combination of MB with MTX significantly reduced the activity of GGT in the hind paw joint but not in the spleen (Figure 4).

### 3.3. Interleukin-17A in Blood Plasma

**Figure 5:**
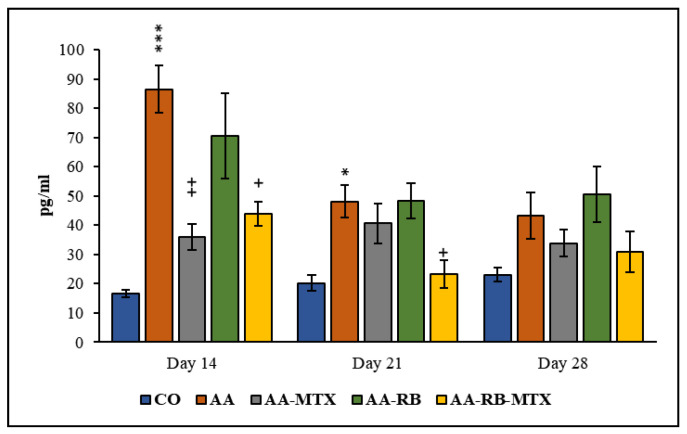
The effect of RB administered alone or in combination with MTX on the levels of IL-17A in blood plasma on days 14, 21, and 28. CO—control animals, AA—adjuvant arthritis controls, AA-MTX—adjuvant arthritis group treated with methotrexate 0.3 mg/kg of b.w. twice a week, AA-RB—adjuvant arthritic animals treated by RB in dosage of 50 mg/kg, AA-RB-MTX—adjuvant arthritic animals treated with RB (50 mg/kg) and 0.3 mg/kg of b.w. twice a week. Results were expressed as mean ± SEM, *n* = 8. Significant difference: *** *p* < 0.001 vs. CO, ++ *p* < 0.01 vs. AA, * *p* < 0.05 vs. CO, + *p* < 0.05 vs. AA.

The concentrations of IL-17A in blood plasma were determined on experimental days 14, 21, and 28. In the model group (AA), IL-17A concentration were markedly enhanced at day 14 and then gradually dropped within the duration of the experiment and were only insignificantly higher at the end of the experiment. Only on day 14 in the MTX group did the plasma levels of this cytokine decrease significantly compared to AA group. The treatment of combination of RB-MTX group showed a significant decrease (Figure 5) in levels of this cytokine on days 14 and 21 when compared to untreated AA rats. Additionally, plasma levels of IL-17A in the combination group of RB-MTX were decreased by 42.6% and 12.7% more efficiently, when compared to MTX monotherapy on days 21 and 28 (Figure 5).

## 4. Discussion

This is the first in vivo study, were the anti-inflammatory effect of the active substance robinin (RB) on the development of adjuvant arthritis (AA) was investigated. We have also examined the combined administration of RB with antirheumatic drug MTX. Adjuvant arthritis is recognized as one of extensively used animal model for studying the series of inflammatory processes that occur in RA and validating novel anti-inflammatory and anti-rheumatic drugs [20,21,26,27]. The key drugs for RA treatment are disease modifying anti-rheumatic drugs (DMARDs), including MTX. MTX is able to decrease synovial, as well as systemic, inflammatory processes, and it improves the function of joints [28,29]. To determine the effectiveness of the substance tested, MTX was chosen as the most commonly used drug in RA. In our previous studies, MTX has been proved as a good standard to examine the efficiency of experimental treatments [28]. Furthermore, MTX is the cornerstone in the therapy of RA either as a single drug or in combination with other drugs to maximize the therapeutic efficacy [30,31,32]. It is little known about the bioavailability of robinin. In previous studies we examined the possible involvement of membrane efflux transporter (P-glycoprotein) in oral absorption of this compound. According to the obtained results P-glycoprotein, located in the intestinal epithelial cells may be responsible for its low oral bioavailability [33]. Robinin is a very safe compound, its LD_50_ is higher than >1 g/kg in mice [34,35]. According to this acute toxicity study, a safe dose of 50 mg/kg of RB was selected for this experimental design.

AA animals have a quick onset and progression of disease with poly-articular inflammation, which is manifested by increased volume of the hind paw and a typical course of loss of body weight [36].

In all AA rats, the body weight gain was significantly lower than in control animals (Figure 2), which may be due to the alterations in the metabolic activities caused by systemic inflammation during AA [37]. Earlier findings suggested that major clinical markers, HPV and body weight gain, are remarkably worsened due to inflammation and were effectively diminished by administration of compounds with anti-oxidative and/or anti-inflammatory properties [38,39,40]. The protective effect of MTX against AA development agrees with previously reported results [23,28,37,40,41] as well as with changes of HPV shown in Figure 3. Combination of RB with MTX did not improve these protective effect of MTX (Figure 2 and Figure 3). Gamma-glutamyl transferase (GGT) is an enzyme found in cell surface of various tissues of the body and considered as one of the pathogenic factors involved in the inflammatory processes. Raised expression and activity of GGT in joint tissue is a good experimental standard for synovial inflammation in collagen-induced arthritis. It is thought that neutralization of GGT with anti-GGT antibody might be novel therapeutic agents for attenuating joint destruction in RA patients [42,43]. In this study, the GGT activity was higher in AA animals than in healthy controls in the spleen and in the joints homogenates (Figure 4). These data resemble the clinical studies of patients suffering RA with raised activity of GGT not only in the urine and serum, but in synovial fluid too [44]. The combination of MTX and RB had a significant effect in lowering the GGT activity in joints of animals (Figure 4A). The effectivity of MTX was marked also in studies with a similar setting and design of the experiment [37,45]. In earlier investigations, we demonstrated a positive agreement between the GGT activity in joint tissue and the hind paw volume of AA animals [46]. Correspondingly, as it is shown in Figure 4A, our previous results [37,45,46] are also in a good agreement with the current observation in changes of HPV (Figure 3).

Suppressing cytokines with natural products has become an important focus in the development of new drugs to treat RA [47]. Inflammatory processes in RA, such as swelling of the synovium of the joint, with subsequent destruction of articular structures are associated with activation of synovial fibroblasts and Th-lymphocytes [48]. In the last few years, various studies figured out that the manifestation of RA is critically dependent on expression of TLR family members expression, which in turn is acknowledged to play a crucial role in T-lymphocytes function and differentiation [49,50,51]. Previous studies have shown the importance of TLR4 and TLR2 function in the pathogenesis of RA. Since the expression of these genes is increased and regulated by proinflammatory cytokines. Activation of TLRs intensifies T-helper lymphocytes (Th17) cell expansion in pro-inflammatory cytokine-dependent signaling, with increased accumulation of interleukin IL-17 [52,53]. IL-17A is the key cytokine of the Th17 population and has been involved in the inflammatory processes of RA. Anti-IL-17 treatments could improve approaches to control the disease chronicity. Several studies have shown the increased concentration of IL-17A and/or Th17 in inflamed joints and blood of the patients suffering RA. It was recently shown a relation of IL-17A concentrations with the disease activity or joint damage [54,55]. Recent understanding of the etiopathogenesis of RA emphasized the role of the cytokine network in the initialization and development of the illness, which has bought a novel class of drugs for RA directly targeting cytokines [56]. Experimental, as well as clinical, proofs exhibit that IL-17A is a therapeutically appropriate target for RA interventions [57]. Multiple hypotheses, including alteration of cytokine profiles, have been proposed to explain the mechanism of MTX efficacy in RA [58]. MTX has been found to influence cytokine production and inhibits the up-regulations of IL-17A in the co-culture of T-cells and fibroblasts. Li et al. (2012) showed that MTX can suppress IL-17 production, which could support its anti-inflammatory activity in the therapy of RA [59]. Luo et al. [60] hypothesized that MTX may exert its therapeutic effect by inhibiting the expression of TLR2 and TLR4. The antioxidant and anti-inflammatory phytochemicals can be used as potential therapeutic agents for arthritis.

In our study, we examined the effect of RB on the plasma concentration of IL-17A during the development of AA. Recent works suggests that IL-17 contributes to RA chronicity and late pathogenic responses through synovial inflammation and hyperplasia [61].

This also applies for AA [62,63,64]. RB alone has no effect on the IL-17A levels in plasma (Figure 5). However, RB in combination with MTX decreased the IL-17A levels in plasma on days 14 and 21 significantly (Figure 5). Our previous results with fatsiphloginum, especially in combination with MTX, are comparable, with results from this study [41]. The anti-inflammatory activity of robinin could be explained also via its metabolites. Administration of robinin to rats resulted in the urinary excretion of kaempferol with smaller amounts of *p*-hydroxyphenylacetic acid. Formation of *p*-hydroxyphenylacetic acid was increased in animals that had previously received a diet containing kaempferol or robinin. The same metabolites were shown to be formed on incubation of robinin with the microflora [65]. The 3-flavonols kaempferol and robinin undergo a cleavage reaction analogous to that reported for quercetin, and these metabolites were reported to have biological activities [66]. Kaempferol as a major metabolite of robinin possesses also anti-inflammatory effects. These might be responsible for anti-inflammatory effects observed after per oral administration of robinin, as well. The in vitro study of Lee et al. (2010) has shown that kaempferol has significantly inhibited cyclooxygenase 1 and 2 (COX2) reaction. Kaempferol and cytokines have been incubated with isolated human hepatocytes, and it was noted that COX2 and inducible NO-synthase levels were decreased [67]. Moreover, kaempferol also suppressed the release of IL-6, IL-1β, IL-18, and TNF-α in lipopolysaccharide plus adenosine triphosphate-induced inflammatory response in cardiac fibroblasts [68]. Probably the beneficial anti-inflammatory activity of RB in combined with MTX might be due to the inhibition of the release of pro-inflammatory cytokines in joints of AA animals.

## 5. Conclusions

In this study, the anti-inflammatory and anti-arthritic activity of robinin (RB) was evaluated for the first time. RB in monotherapy was not efficient in reducing any of parameters measured. The beneficial properties of MTX monotherapy were not enhanced by the co-administration of RB on CBW and HPV. However, the combination of MTX-RB was more effective in decreasing the activity of GGT in the hind paw joint and in lowering the plasmatic level of IL-17A than MTX alone. However, further investigations on the mechanism of anti-inflammatory and anti-arthritic action, as well as the oral pharmacokinetic profile of RB, are needed for better understanding of robinin efficacy in RA. Importantly, RB is a very safe compound and, hence, potentially suitable for combination regimens.

## Figures and Tables

**Table 1:** Experimental design.

Group	Treatment and Active Substance	Posology
Group 1: Healthy controls (CO)	vehiculum	0.5 mL
Group 2: AA untreated	vehiculum	0.5 mL
Group 3: AA + treatment	Methotrexate (MTX)	0.3 mg/kg of b.w. twice a week
Group 4: AA + treatment	Robinin (RB)	50 mg/kg of b.w. daily
Group 5: AA + treatment	Robinin+MTX (RB-MTX)	50 mg/kg + 0.3 mg/kg of b.w. twice a week

AA—adjuvant arthritis, MTX—methotrexate, b.w.—body weight.

## Data Availability

Data from this study are available here: https://drive.google.com/file/d/109aUyrDB3Fy62avxtc1GcCqSUyyj3bNj/view?usp=sharing (accessed on 26 February 2021).
